# Continuous assessment of tissue perfusion using quantitative indocyanine green fluorescence imaging during controlled hypo- and reperfusion

**DOI:** 10.1007/s00464-025-11905-z

**Published:** 2025-07-02

**Authors:** Frederik Thørholm Andersen, Jacob Petersen, Alexander Emil Kaspersen, Mads Vikkelsø Pedersen, Gustav Singer, Mads Holst Aagaard Madsen, Morten Asp Vonsild Lund, J. Michael Hasenkam, Troels Lading

**Affiliations:** 1 Department of Cardiothoracic and Vascular Surgery, Aarhus University Hospital, Palle Juul-Jensens Boulevard 69, 8200 Aarhus N, Denmark; 2 Department of Clinical Medicine, Faculty of Health, Aarhus University, Århus, Denmark; 3 Perfusion Tech ApS, Copenhagen, Denmark

**Keywords:** Fluorescence imaging, Indocyanine green, Transit time flow measurements, Perfusion, Hypoperfusion, Reperfusion, Kidney

## Abstract

**Background:**

Indocyanine green fluorescence imaging (ICG-FI) is a novel tool for continuous assessment of tissue perfusion. However, consensus on the optimal methodology is lacking.  It was hypothesized that quantitative ICG-FI parameters based on continuous micro-dosing correlate with corresponding transit time flow measurements in the functional renal end artery.  This study aimed to examine the feasibility of continuous, quantitative ICG-FI for detecting perfusion changes (hypoperfusion, reperfusion) in porcine kidneys.

**Methods:**

Renal perfusion was assessed using continuous, quantitative ICG-FI under controlled renal artery flow adjustments (no, partial, full, partial, and no occlusion) in ten healthy female pigs. Four 0.008 mg/kg ICG micro-doses were administered with 60-s intervals for each flow adjustment. As a reference, simultaneous renal artery transit time flow probe measurements were recorded. ICG-FI parameters were extracted by PerfusionWorks^®^, providing surrogate markers for perfusion, and correlated to the reference renal artery transit time flow measurements using linear regression modeling.

**Results:**

During hypoperfusion, mean flow decreased from 277 to 139 to 0 ml/min in the no, partial, and full occlusion steps, respectively. *F*_ingress_ (*R*^2^ = 79%), *F*_max_ (*R*^2^ = 79%), and slope (*R*^2^ = 78%) correlated with flow. During reperfusion, mean flows increased from 0 to 169 to 240 ml/min in the full, partial, and no occlusion steps, respectively, and *F*_ingress_ (*R*^2^ = 71%), *F*_max_ (*R*^2^ = 69%), and slope (*R*^2^ = 79%) also correlated with flow. Overall, slope exhibited the strongest correlation (*R*^2^ = 54%). *T*_ingress_, *T*_½max_, time ratio, and *T*_max_ exhibited poor correlations with flow.

**Conclusion:**

Continuous assessment of renal perfusion was technically feasible. In this kidney model, the combined ICG-FI parameter, slope, and the intensity-dependent parameters *F*_ingress_ and *F*_max_ exhibited convincing correlation with flow, appearing promising for the continuous assessment of tissue perfusion. Time-related parameters did not correlate with flow in this specific model, likely due to the kidney's unique vascular characteristics.

**Graphical abstract:**

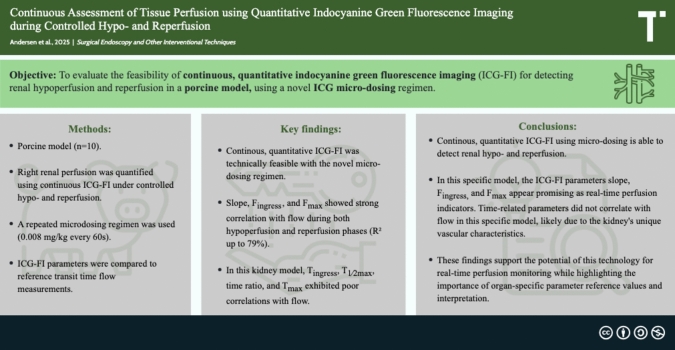

Identification and timely intervention of ischemia is key in most surgical procedures [1]. Therefore, a sophisticated method to provide real-time information on tissue perfusion may reduce the risk of ischemia. The ideal method should be safe, non-invasive, offer a high sensitivity and specificity, and enable continuous, real-time monitoring of tissue perfusion [2–4]. Near-infrared fluorescence imaging using indocyanine green (ICG-FI) is a well-known method for real-time assessment of tissue perfusion. It offers substantial clinical value by allowing early identification of hypoperfusion. Thereby, steps can be taken timely to prevent postoperative complications such as ischemia and necrosis. It has previously been used in urology [5], plastic surgery [6, 7], gastrointestinal surgery [8–11], cardiothoracic surgery [12, 13], vascular surgery [14, 15], and neurosurgery [16, 17].

Upon intravenous injection, indocyanine green (ICG) binds to plasma proteins, restricting the fluorophore to the intravascular compartment during its plasma half-life of 3–5 min [18]. Exposure to near-infrared light excite ICG and facilitate the emission of fluorescence, which enables visualization and assessment of tissue perfusion [19]. The underlying principle is the fluorescence intensity-signal being proportional to the amount of ICG transiting through the vascular bed, and thus a surrogate marker for perfusion.

The qualitative, conservative ICG-FI assessment is performed by visually interpreting the fluorescence intensity-signals emitted from the tissues, using a colored overlay on a screen. It is the most frequently used approach to evaluate tissue perfusion based on ICG. However, since tissues accumulates ICG during repeated administration, adequate ICG clearance is required for meaningful visual interpretation and limits the number of possible evaluations. Moreover, the maximum recommended human dose is 2 mg/kg ICG per day limits its utility, and factors such as camera distance, dosage, and white-light contamination leave the qualitative assessment susceptible to bias [20]. On the other hand, the novel, quantitative approach is performed objectively, using newly available image analysis software. The method is quantitative since the computational signal processing of the fluorescence intensity peaks, derived from time-intensity curves, provides a numerical output in terms of ICG-FI parameters rather than displaying shades of gray or green [21, 22]. This allows for an objective assessment at a much lower ICG dosage, potentially enabling continuous quantitative assessment of tissue perfusion without exceeding the maximum daily dosage.

ICG-FI parameters can be intensity-dependent, time-related, or combined and offer an opportunity to establish thresholds of sufficient perfusion in a clinical context [7]. Yet, consensus is still lacking on which ICG-FI parameter to favor. Preclinical investigations have revealed that both conditional and patient-specific factors exert various influence on each individual group of ICG-FI parameters [20, 23, 24]. Intensity-dependent parameters are more commonly used and thus more validated, but time-related parameters appear less susceptible to intra- and interpatient variability [22]. Clinical studies have explored the clinical performance of specific cut-off values in detecting inadequate tissue perfusion [7, 25–28], but only few studies have examined the feasibility of a repeated, high-frequency, low-dose ICG bolus regimen adapted for continuous tissue perfusion monitoring [20, 29]. Recently, a novel, proprietary concept was devised which combine the quantitative software PerfusionWorks^®^ (Perfusion Tech ApS, Copenhagen, Denmark) with a repeated, high-frequency, low-dose ICG bolus regimen, potentially allowing for continuous monitoring of tissue perfusion. However, it is still unknown which ICG-FI parameter represent tissue perfusion most precisely during continuous assessment of organ perfusion, and thus, which ICG-FI parameter cut-off values should be based on [30, 31].

We hypothesized that ICG-FI parameters exhibit correlation with corresponding transit time flow probe measurements in a functional end artery, being applicable as surrogate markers for tissue perfusion. We aimed to assess the feasibility of quantitative ICG-FI by analyzing the correlation of intensity-dependent, time-related, and combined ICG-FI parameters with transit time flow probe measurements in a functional end artery, and to identify the optimal parameters to disclose hypoperfusion and reperfusion in a continuous perfusion assessment context.

## Materials and methods

This experimental animal study was conducted at Department of Clinical Medicine, Aarhus University, Aarhus, Denmark. Each pig was subjected to quantitative ICG-FI and simultaneous transit time flow measurements, which enabled each pig to serve as its own control, eliminating the need for control groups undergoing sham procedures, randomization, and blinding.

### Animals

Ten healthy, female mixed Duroc and Danish Landrace-Yorkshire pigs (aged 16–20 weeks) with a body weight of 60 kg were enrolled for this experiment. All pigs were bred under standard laboratory conditions at Aarhus University Experimental Animal Farm, Aarhus, Denmark and were subjected to enrichment and daily training. Prior to transportation from the farm to the operating room, the animals were premedicated with an intramuscular injection of 0.1 ml/kg Zoletil vet-mixture (zolazepam, tiletamin, xylazine, ketamine, butorphanol, and methadone). Upon arrival to the laboratory, peripheral venous catheters were placed in both ears and endotracheal intubation was performed. Mechanical ventilation was applied to all pigs and the settings were adjusted to achieve normoventilation with a FiO2 level of 40%. The animals were kept anesthetized during the entire experiment with continuous intravenous infusion of 3.7 mg/kg/h propofol and 6.2 μg/kg/h fentanyl. Relaxation was maintained by intravenous infusion of 3.3 mg/kg/h rocuronium if needed. Intravenous injections of 10 mg/kg ketamine hydrochloride were intermittently given to avoid shivering. Invasive blood pressure was monitored by catheterization of the right femoral artery, while the jugular vein was catheterized for intraoperative ICG injections. Hemodynamic stability was ensured by means of fluid therapy (0.9% NaCl), atropine, noradrenaline, and Trendelenburg position if needed. Invasive blood pressure monitoring, pulse oximetry, and electrocardiography was continuously applied to monitor the hemodynamic status.

### Ethics

The study was performed in accordance with the National Guidelines for Experimental Animal Research and European legislation (Directive 2010/63/EU) and the protocol was approved by the Danish Animal Experiments Inspectorate (2022-15-0201-01153). Reporting was done in compliance with the ARRIVE 2.0 guidelines [32]. All animal management was performed under the supervision of in-house research veterinarians. The animals were fully anesthetized during the entire experiment and euthanized under continued anesthesia at the end of each experimental day by intravenous administration of an overdose of pentobarbital.

### Sample size justification

Continuous ICG-FI-based perfusion assessment is not currently part of standard clinical practice, and no prior studies have provided data that would allow for a conventional power calculation for this method. Consequently, the sample size was determined based on findings from a pilot study and a comprehensive review on sample size considerations in feasibility and pilot studies [33]. This approach was further guided by ethical considerations aligned with the 3Rs principles for animal research [34], as well as the research group’s extensive experience with animal studies. Based on these considerations, a sample size of 10 pigs was deemed sufficient to meet the study objectives.

### Experimental setup

A midline laparotomy was performed, and the right kidney was exposed. Since the porcine renal artery shows an anatomical pattern distinct from its human counterpart by distally splitting into several branches, ultrasound with color Doppler was used to identify all peripheral branches. A transit time ultrasonic flow probe (SonoTT Vascular Probes with handle (Em-tec GmbH, PSG^®^ Dover Corporation, Oakbrook Terrace, IL, USA)) was placed on the right renal artery at its origin from the abdominal aorta with an upstream position to all arterial branches. The probe size was carefully chosen on a case-by-case basis to ensure proper vessel fit with the dimensions of each vessel. Motion artifacts related to manual clamping and flow tracing were minimized by applying mechanical fixation or a “no-touch” technique during each occlusion step.

**Fig. 1:**
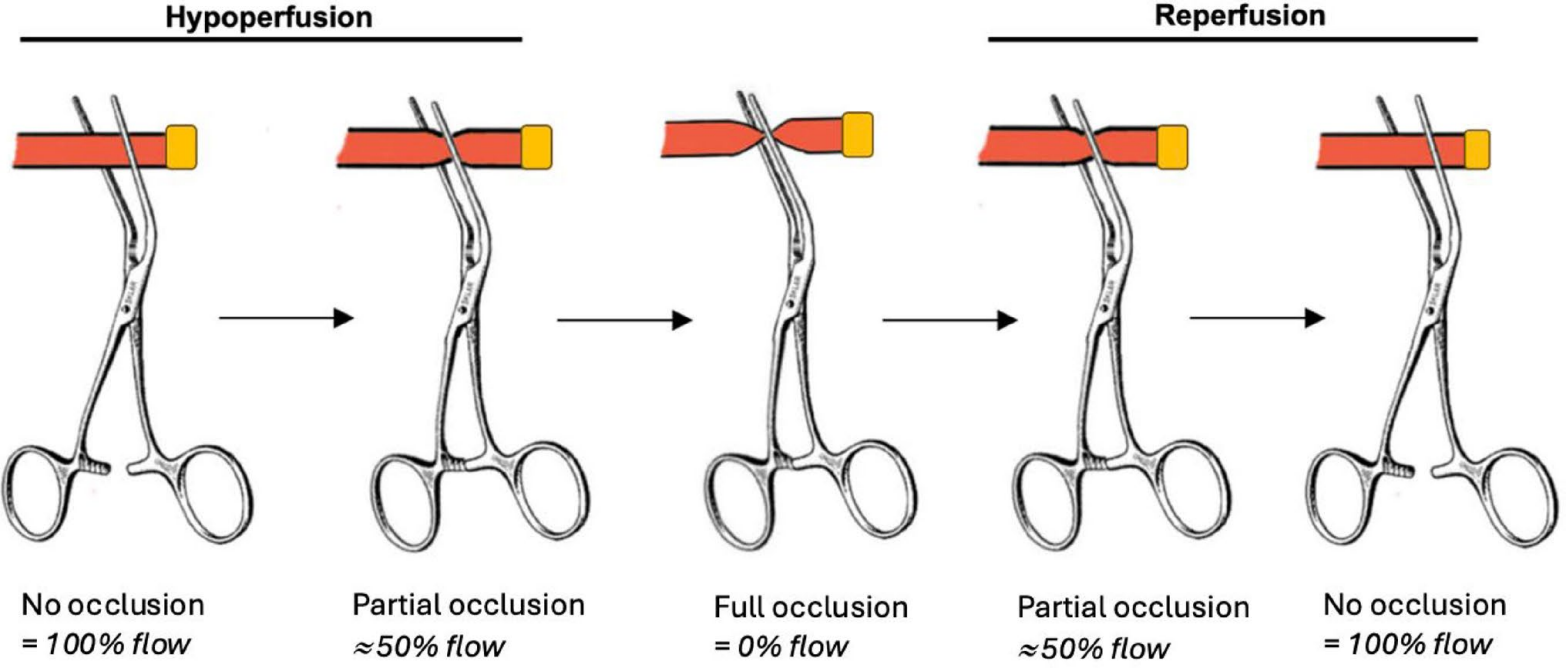
Graphical illustration of each step of occlusion of the renal artery during the hypoperfusion and reperfusion phases under continuously monitoring of flow. The yellow box illustrates the transit time flow probe placed downstream of the clamp (Color figure online)

ICG-FI and transit time flow probe measurement were performed simultaneously in five steps (no occlusion, partial occlusion, full occlusion, partial occlusion, and no occlusion), each step being 240 s, with varying occlusion of the right renal artery to simulate renal hypoperfusion (no to partial to full occlusion) and renal reperfusion (full to partial to no occlusion), respectively. Renal artery occlusion was performed using an angled 55° DeBakey Peripheral Vascular Clamp (Jarit^®^, Integra LifeSciences, Princeton, N.J., USA) placed upstream of the flow probe (Fig. 1). The vascular clamp was occluded by hand to a 50% reduction in flow during the partial occlusion steps and 100% reduction in the full occlusion step. For each occlusion step, a single transit time flow measurement was recorded with the SonoTT Flowlab™ system (Em-tec GmbH, PSG^®^ Dover Corporation, Oakbrook Terrace, IL, USA).

The kidney was selected as an optimal model for this feasibility study due to several advantageous characteristics. Its blood supply consists of a single large vessel that is easily accessible and located close to the aorta, allowing for precise blood flow control through clamping while simultaneously measuring flow via ultrasound Doppler. This anatomical configuration enabled us to create controlled, reproducible conditions of hypoperfusion and reperfusion, necessary for validating the correlation between ICG-FI parameters and actual blood flow. However, we acknowledge that the unique vascular anatomy of the kidney, characterized by its short vascular pathway from the aorta and its robust autoregulatory mechanisms, creates a specific context for interpreting our findings, particularly regarding time-related metrics.

### Quantitative indocyanine green fluorescence imaging

ICG-FI was used to continuously quantify ICG fluorescence intensity over time in each occlusion step, using an ICG micro-dosing regimen adapted for continuous monitoring. For every 60 s, corresponding to a measurement cycle, an ICG micro-dose of 0.008 mg/kg ICG dye (Verdye^®^ 5 mg/ml, Diagnostic Green GmbH, Munich, Germany) was administered as a 1 ml bolus, followed by a 3 ml saline flush at a rate of 75 ml/min into the jugular vein. This was accomplished automatically using a research version of PerfusionWorks^®^ (Perfusion Tech ApS, Copenhagen, Denmark) and a commercially available programmable infusion pump CE marked for laboratory use (Legato 110, KD Scientific, Massachusetts, USA). This dosing regimen was selected based on a preclinical feasibility study performed in visceral organs, aiming to minimize cumulative ICG accumulation while maintaining adequate signal-to-noise ratios [20]. The study implemented a repeated micro-dosing regimen of 0.008 mg/kg ICG with 60-s measurement intervals and found that tissue accumulation of ICG over time did not interfere with the quantification process. Thus, the short plasma half-life of ICG combined with the micro-dosing regimen were anticipated to limit re-injection variability and maintain stable baseline fluorescence levels during repeated measurements.

The surgical imaging was performed using a QUEST SPECTRUM^®^ 3 (Olympus Europa SE & Co. KG., Hamburg, Germany). The following camera characteristics was applied for all measurements; mode—800 nm (supports the use of ICG); laser exposure—4000; visible exposure—10,000; laser gain—0; visible gain—0. The 2 × 2 screen viewing mode was used, presenting one of the following screens in each quadrant: Red/Green/Blue (natural image that can be seen by the eye in color), Near-infrared (fluorescent response in grayscale), Overlay (overlay of fluorescent image in green with the color image), and Gradient (overlay of the fluorescent response in color (blue to red) on the color image in greyscale). The imaging system offers a function that saves personally preferred settings, which allowed us to operate with constant settings in all experiments. To avoid variation in setup, the Quest SPECTRUM Ring Light was positioned with a 20 cm distance from camera lens to tissue and directed perpendicularly to the examined tissue plane, and the operating room was cleared for ambient light before all measurements. Fluorescence quantification was performed using a research version of the commercially available ICG image analysis software, PerfusionWorks^®^. The software allows for the selection of multiple size-adjustable regions of interest (ROIs) and provides perfusion status information in terms of ICG-FI parameters for each anatomical area of interest. The software is designed to facilitate real-time, intraoperative quantitative perfusion assessment and was operated through an external laptop connected to the video input of the surgical platform. In our setting, a target ROI was placed retrospectively for the parameter analysis to select an optimal target tissue, comparable between pigs. This was defined by a parenchymal anatomical field near the renal hilus.

**Fig. 2:**
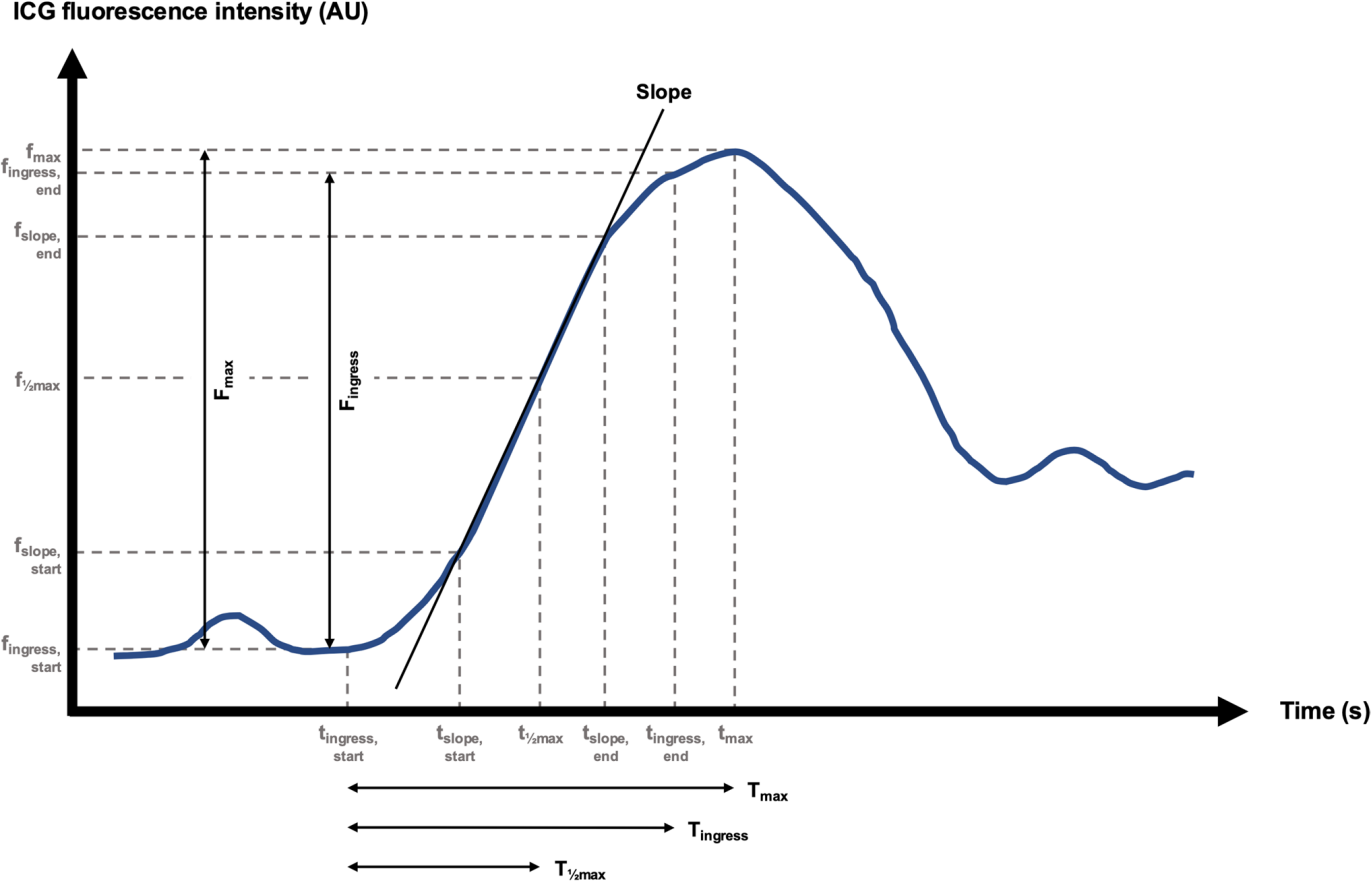
Graphic illustration of time-intensity curve and included PerfusionWorks^®^ parameters (black) and values used for calculation (gray). The blue line illustrates the indocyanine green fluorescence intensity over time. AU: arbitrary units; *f*_ingress, start_: onset of fluorescence intensity increase from background fluorescence intensity; *f*_ingress, end_: end of fluorescence intensity increase; *F*_ingress_: change in fluorescence intensity from *f*_ingress, start_ to *f*_ingress, end_; *f*_max_: maximum fluorescence intensity; *F*_max_: change in fluorescence intensity from *f*_ingress, start_ to *f*_max_; ICG: indocyanine green; *t*_ingress, start_: time of *f*_ingress, start_; *t*_ingress, end_: time of *f*_ingress, end_; *T*_ingress_: time from *t*_ingress, start_ to *t*_ingress, end_; *t*_max_: time of *f*_max_; *T*_max_: time from *t*_ingress, start_ to *t*_max_; *t*_½max_: time of half maximum fluorescence intensity; *T*_½max_: time from *t*_ingress, start_ to *t*_½max_ (Color figure online)

The software generates an ICG fluorescence time-intensity curve based on the selected ROI from which the data is extracted. The software determines the output based on the configuration of the fluorescence signal peak, quantifying well-established ICG-FI parameters described in the literature [35]. Time-related parameters determined by PerfusionWorks^®^ included *T*_max_, *T*_½max_, time ratio (*T*_½max_/*T*_max_), and T_ingress_. Intensity-dependent parameters included *F*_max_, and *F*_ingress_. Lastly, the combined time-intensity parameter slope was included. Each ICG-FI parameter examined in this study is defined in Table 1 and graphically illustrated in Fig. 2. These parameters reflect different physiological aspects of tissue perfusion. Time-related parameters like T_1/2max_ primarily represent the speed at which blood flows to an organ, reflecting the cumulative vascular resistance and the length the blood must move through the vascular tree. Physiologically, T_1/2max_ is independent of absolute fluorescence intensities, making it reliable for assessing microcirculatory function across various tissues and imaging conditions. Meanwhile, the slope parameter represents both speed and volume of blood flow, quantifying both the rate of vascular filling and the total perfusion volume. A steeper slope indicates rapid inflow and robust perfusion, while a reduced slope suggests vascular insufficiency. These parameters are complementary when assessing tissue perfusion during fluorescence-guided imaging.

**Table 1:** Indocyanine green fluorescence imaging parameter definitions

*Time-related parameters*	
*T*_max_	Time from time of onset of fluorescence intensity increase from background fluorescence intensity (*t*_ingress, start_) to time of maximum fluorescence intensity (*t*_max_)
*T*_½max_	Time from time of onset of fluorescence intensity increase from background fluorescence intensity (*t*_ingress, start_) to time of half maximum fluorescence intensity (*t*_½max_)
Time ratio	*T*_½max_/*T*_max_
*T*_ingress_	Time from time of onset of fluorescence intensity increase from background fluorescence intensity (*t*_ingress, start_) to time of end of fluorescence intensity increase (*t*_ingress, end_)
*Intensity-dependent parameters*	
*F*_max_	Change in fluorescence intensity from onset of fluorescence intensity increase from background fluorescence intensity (*f*_ingress, start_) to maximum fluorescence intensity (*f*_max_)
*F*_ingress_	Change in fluorescence intensity from onset of fluorescence intensity increase from background fluorescence intensity (*f*_ingress, start_) to end of fluorescence intensity increase (*f*_ingress, end_)
*Combined time-intensity parameter*	
Slope	The maximum increase in fluorescence intensity per second

### Statistical analysis

ICG fluorescence time-intensity curves for the four measurement cycles within each occlusion step were visually inspected to remove potential outliers, defined as curves being irregular compared with its corresponding curves. Adjustment of the time-intensity curves was accomplished by subtracting the mean ICG fluorescence intensity of the first ten baseline measurements from each measurement cycle in each occlusion step for each pig. The ICG-FI parameters were determined by PerfusionWorks^®^ and averaged for each occlusion step for each pig. Results are presented as mean with 95% confidence interval (CI).

Mean flow and ICG-FI parameters were compared between occlusion steps by mixed effects models with occlusion step as fixed effect and pig as random effect. Models were fitted using restricted maximum likelihood [36], and the correction method of Kenward and Roger was applied to reduce small sample bias [37]. Normality of residuals, the variances of the residuals, and random effects were checked by inspection of quantile plots of residuals and best linear unbiased predictors, respectively, and all parameters and flow values were analyzed correspondingly. Post hoc pairwise comparisons between occlusion steps were made using two-tailed least square means pairwise *t*-test.

Linear regression modeling was performed on mean values for each occlusion step to assess correlation between flow and each ICG-FI parameter. Estimate, R2-value, and adjusted R2-value, taking small sample bias into account, was estimated, and difference in parameter estimate was tested. Modeling was performed by including all occlusion steps in the analysis and individually for the hypoperfusion and the reperfusion phase.

Time-related parameters (*T*_max_, *T*_½max_, time ratio, and *T*_ingress_) during full occlusion were excluded from linear regression analysis as the absence of perfusion produced flat time-intensity curves without measurable peaks. To ensure consistency the study software was programmed to perform identical best-fit analysis after each ICG bolus regardless of occlusion status. Including these data would have introduced bias from automated processing of noise-related signal oscillations rather than actual perfusion dynamics, compromising the linear regression model's validity.

All models were developed with support from the Biostatistical Advisory Service (BIAS) at Aarhus University. Tests were two-tailed and interpreted at a statistical significance level of 0.05. Statistical analyses were performed using SAS^®^ Enterprise Guide^®^ software, version 7.1 (SAS Institute Inc., Cary, NC, USA).

## Results

Ten pigs successfully underwent simultaneous ICG-FI and transit time flow measurements. However, one pig did not complete the transit time flow measurement during partial occlusion in the reperfusion phase due to a technical error. All animals maintained stable hemodynamic conditions throughout the surgical procedure.

### Transit time flow measurements

**Fig. 3:**
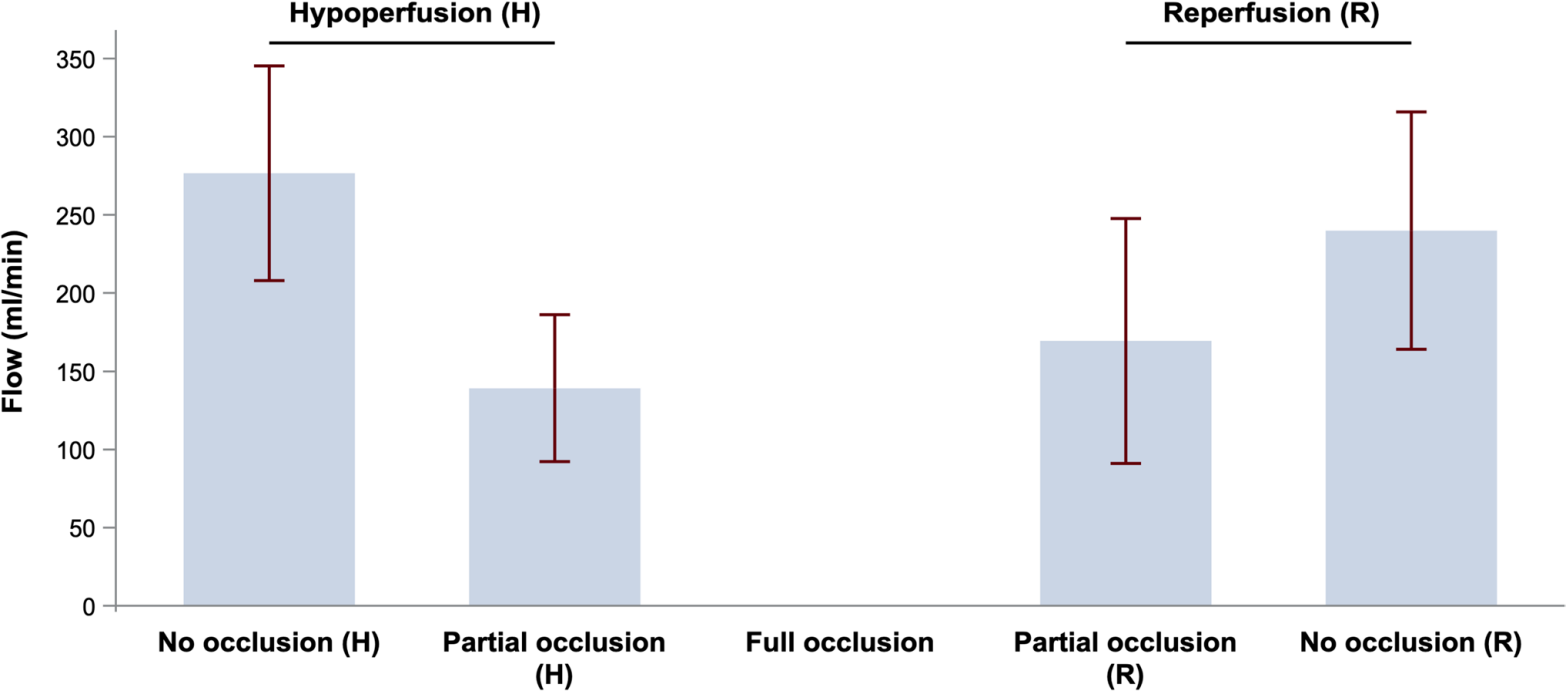
Mean renal artery flow with 95% confidence intervals derived from transit time ultrasonic flow measurements for each occlusion step in the hypoperfusion, full occlusion, and reperfusion phases

Mean flow values with 95% CI of ten pigs in each occlusion step of the hypoperfusion and reperfusion phase are presented in Fig. 3. In the hypoperfusion phase, the mean flow through the renal artery significantly decreased by 137.4 ml/min from no occlusion to partial occlusion (mean [95% CI]: 276.6 [207.9–345.3] vs 139.2 [92.3–186.0] ml/min, *p* < 0.001). Furthermore, the mean flow significantly decreased to no flow from partial occlusion to full occlusion (*p* < 0.001). From full occlusion to partial occlusion, initiating in the reperfusion phase, the mean flow (95% CI) significantly increased to 169.3 (91.0–247.6) ml/min (only based on nine pigs), *p* < 0.001. During the reperfusion phase, the flow further increased by 70.5 ml/min from partial to no occlusion, reaching a mean flow (95% CI) of 239.8 (163.9–315.8) ml/min, *p* = 0.104. There was no statistical difference between the respective occlusion steps in the hypoperfusion vs the reperfusion phase.

### Feasibility of indocyanine green fluorescence imaging

In ten pigs, ICG-FI was successfully applied using the PerfusionWorks^®^ software and was deemed feasible when using the novel repeated, high-frequency, low-dose ICG bolus regimen, resulting in a total infusion of 0.16 mg/kg ICG for each pig. In total, one measurement cycle in two pigs was excluded due to poor image quality, seven measurement cycles spread over four pigs were excluded due to an irregular curve shape compared to the corresponding curves in the same pig, and one measurement cycle was excluded due to signal noise. Thus, 190 measurement cycles out of 200 were included for parameter analysis.

**Fig. 4:**
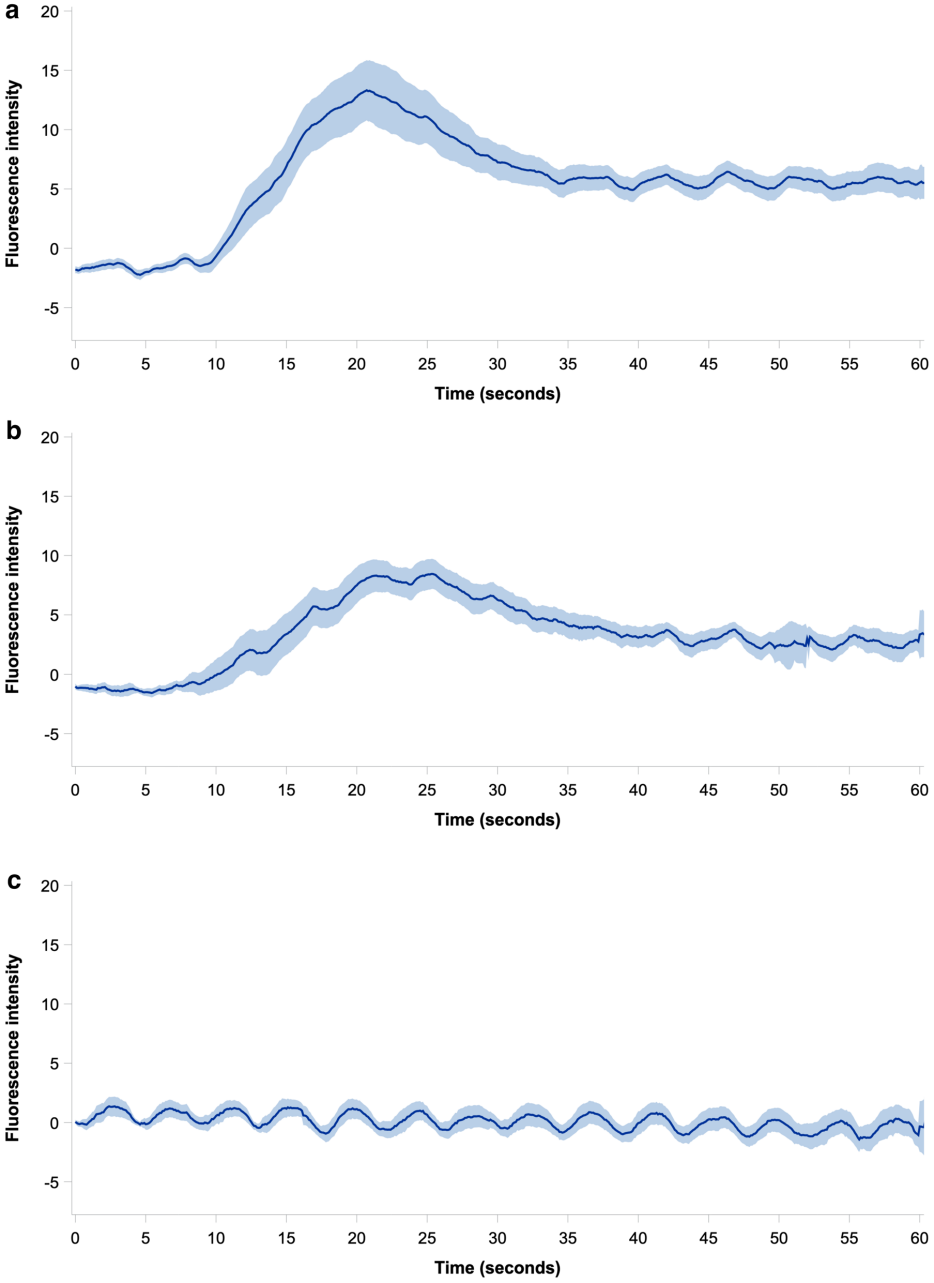

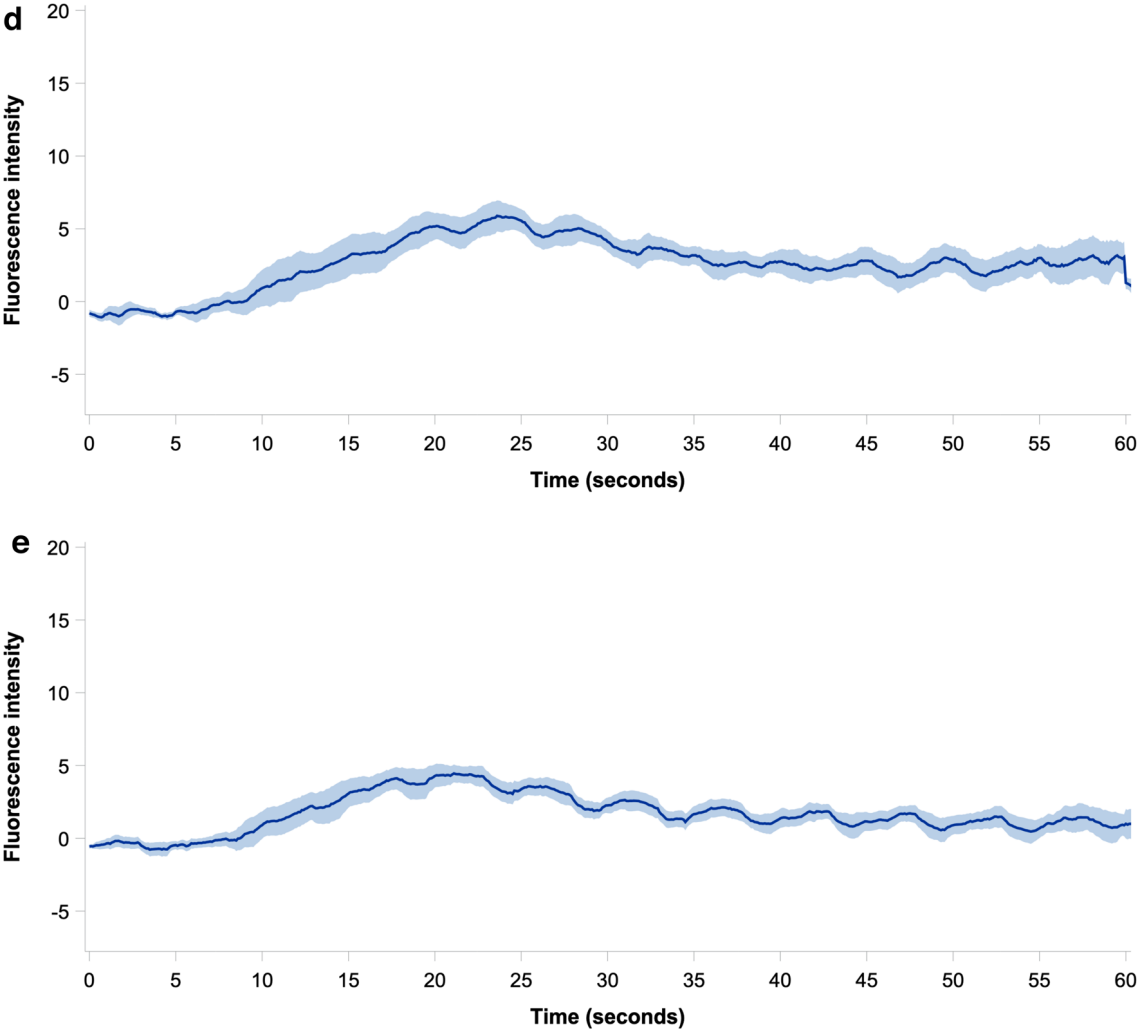
Mean baseline subtracted time-intensity curves (black line) of all measurement cycles within the respective occlusion step with 95% confidence intervals (shaded area) derived from indocyanine green fluorescence imaging for **a** no occlusion (hypoperfusion phase), **b** partial occlusion (hypoperfusion phase), **c** full occlusion, **d** partial occlusion (reperfusion phase), and **e** no occlusion (reperfusion phase) (Color figure online)

**Fig. 5:**
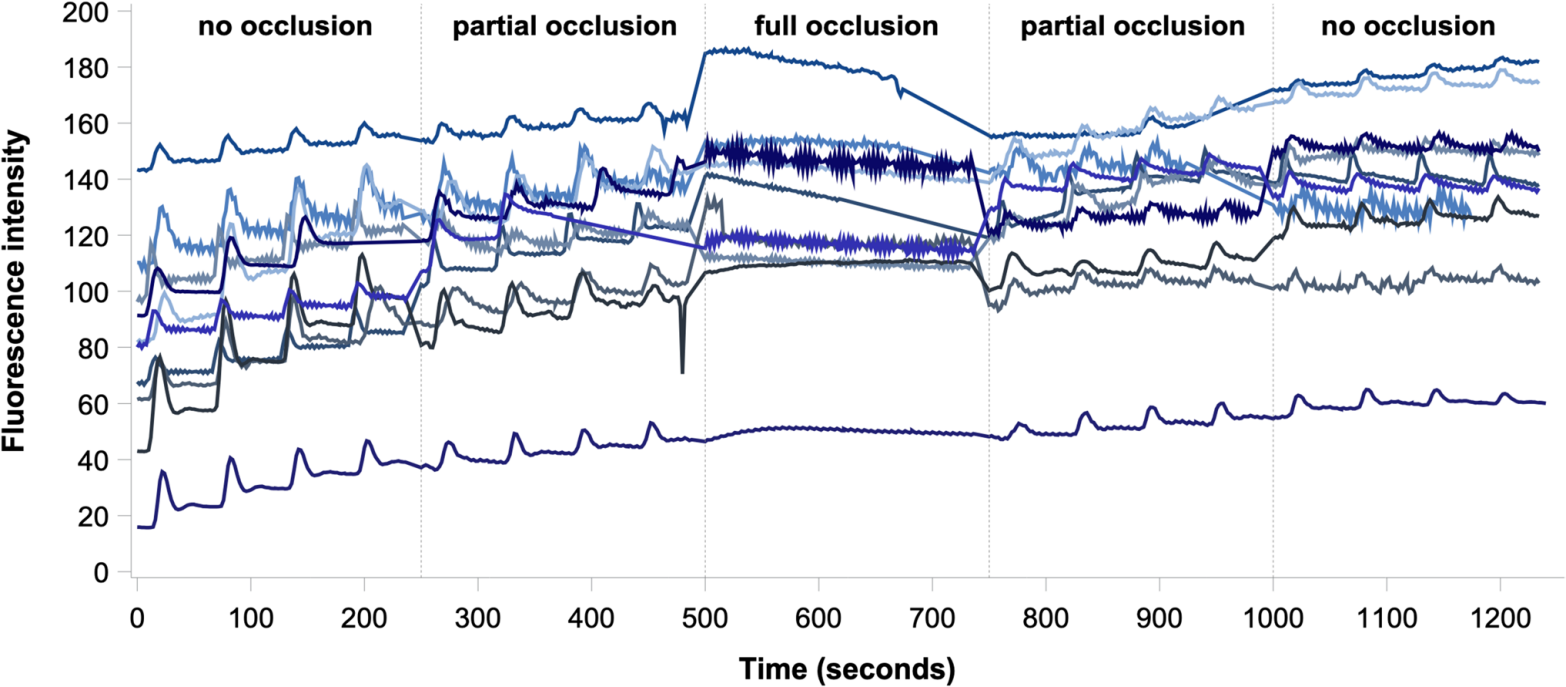
Unadjusted time-intensity curves derived from indocyanine green fluorescence imaging for each occlusion step in the hypoperfusion, full occlusion, and reperfusion phases for each pig (Color figure online)

The mean adjusted ICG fluorescence intensity in each occlusion step is presented as a function of time with 95% CI in Fig. 4, while the unadjusted time-intensity curves for each pig is shown in Fig. 5. Mean values of the ICG-FI parameters slope, *T*_ingress_, *F*_ingress_, *T*_½max_, time ratio, *T*_max_, and *F*_max_ are presented in Table 2. Both slope, *F*_ingress_, and *F*_max_ decreased during hypoperfusion from no to partial occlusion (*p* = 0.043, *p* = 0.026, and *p* = 0.023, respectively) and from partial to full occlusion (all *p* < 0.001), and increased during reperfusion from full to partial occlusion (*p* = 0.010, *p* < 0.001, and *p* < 0.001, respectively) but did not change significantly from partial to no occlusion. Both slope, *F*_ingress_, and *F*_max_ were significantly lower in the no occlusion step during reperfusion than during hypoperfusion (*p* < 0.001) but not different in the partial occlusion steps. No statistical difference between occlusion steps was found for the time-related parameters (*T*_ingress_, *T*_½max_, time ratio, or *T*_max_) after adjustment using Kenward-Roger correction.

**Table 2:** Mean indocyanine green fluorescence imaging derived parameters with 95% confidence intervals for each occlusion step in ten pigs

Parameter	Hypoperfusion		Reperfusion			Mixed effect model *P* value
	No occlusion	Partial occlusion	Full occlusion	Partial occlusion	No occlusion	
Slope (intensity/s)	2.91 (2.08–3.73)	1.97 (1.42–2.52)	0.10 (0.04–0.15)	1.21 (0.72–1.69)	1.15 (0.73–1.57)	< 0.001
*T*_ingress_ (s)	9.22 (7.94–10.51)	9.42 (8.26–10.58)	–	10.33 (8.40–12.26)	9.19 (7.41–10.96)	< 0.001
*F*_ingress_ (intensity)	16.56 (11.75–21.38)	11.80 (9.28–14.31)	0.51 (0.24–0.77)	7.40 (5.63–9.17)	6.09 (4.86–7.32)	< 0.001
*T*_½max_ (s)	5.87 (4.71–7.04)	5.58 (4.93–6.23)	–	6.54 (5.04–8.03)	5.97 (4.56–7.38)	< 0.001
Time ratio	0.55 (0.51–0.59	0.51 (0.46–0.56)	–	0.51 (0.46–0.55)	0.54 (0.51–0.58)	< 0.001
*T*_max_ (s)	10.65 (9.08–12.23)	11.14 (9.72–12.56)	–	13.29 (9.80–16.77)	10.64 (8.69–12.59)	< 0.001
*F*_max_ (intensity)	17.52 (12.34–22.71)	12.39 (9.79–14.99)	0.79 (0.40–1.17)	8.87 (6.06–9.69)	6.59 (5.38–7.80)	< 0.001

Data presented as mean (95% confidence interval)

### Indocyanine green fluorescence imaging derived parameters and flow correlations

**Fig. 6:**
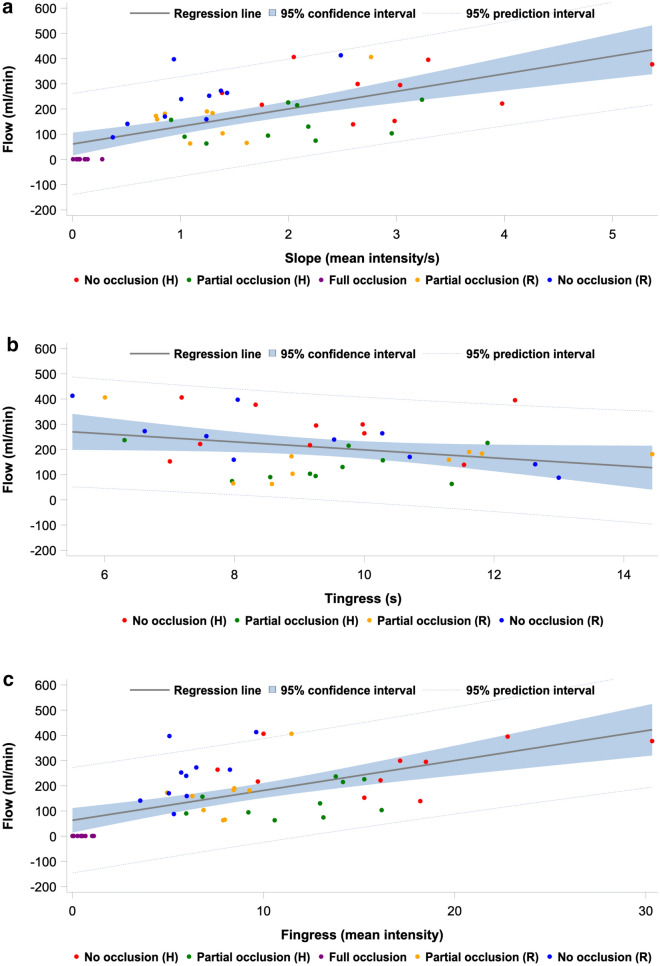

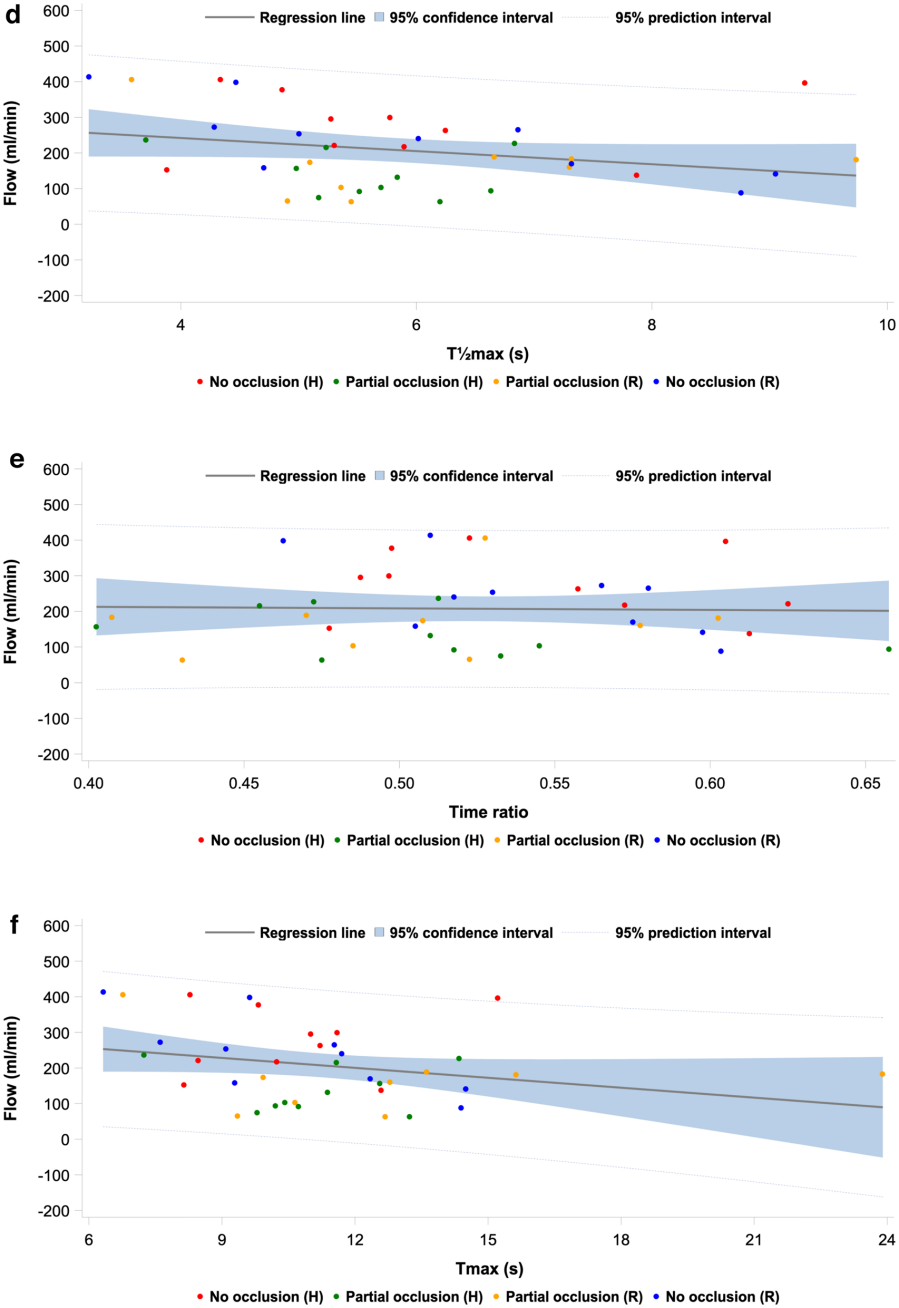

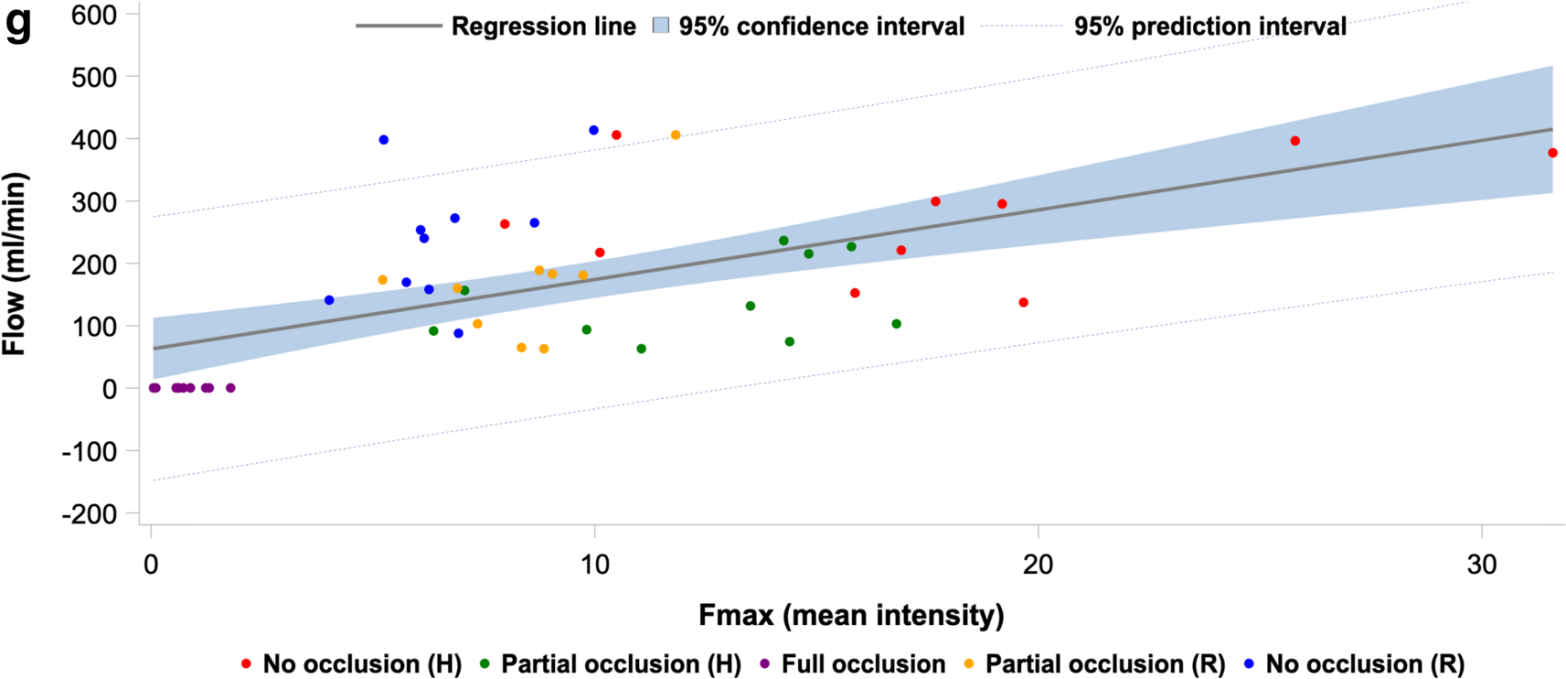
Linear regression plot modeling plot of flow as a function of the ICG-FI parameter **a** slope, **b** *T*_ingress_, **c** *F*_ingress_, **d** *T*_½max_, **e** time ratio, **f** *T*_max_, and **g** *F*_max_ for each pig in each occlusion step, regression line with 95% confidence interval, and 95% prediction interval

Correlation estimate, *R*^2^-value, adjusted *R*^2^-value, and *p* value obtained from linear regression analysis of flow and each of the seven perfusion parameters from the PerfusionWorks^®^ analysis of the ICG-FI time-intensity data for all occlusion steps, the hypoperfusion phase, and the reperfusion phase, respectively, are presented in Table 3. Figure 6a–g show the corresponding linear regression line of the models with corresponding 95% CI and 95% prediction interval. Overall, the combined time-intensity parameter slope exhibited the highest correlation (*R*^2^ = 54%), while the intensity-dependent parameters *F*_ingress_ and *F*_max_ also exhibited high correlations with flow (*R*^2^ = 50% and 48%, respectively). The time-related parameters *T*_ingress_, *T*_½max_, time ratio, and *T*_max_ generally exhibited low correlations. In the hypoperfusion phase, the highest correlations were exhibited by *F*_ingress_ (*R*^2^ = 79%) and *F*_max_ (*R*^2^ = 79%), but this was not significant. In the reperfusion phase, slope exhibited the highest correlation (*R*^2^ = 79%), but also *F*_ingress_ (*R*^2^ = 71%) and *F*_max_ (*R*^2^ = 69%) correlated well with flow. Generally, the parameters displayed higher correlations when analyzed in each phase individually (hypoperfusion phase, reperfusion phase).

**Table 3:** Linear regression results for all indocyanine green fluorescence imaging derived parameters in all occlusions steps, the hypoperfusion phase, and the reperfusion phase

Parameter	Overall				Hypoperfusion				Reperfusion			
	Estimate	R^2^ (%)	aR^2^ (%)	P	Estimate	R^2^ (%)	aR^2^ (%)	P	Estimate	R^2^ (%)	aR^2^ (%)	P
Slope (intensity/s)^a^	92.0	54.1	51.0	< 0.001	24.2	78.1	75.6	0.126	103.8	79.2	76.7	< 0.001
T_ingress_ (s)^b^	− 16.5	11.2	3.6	0.051	0.6	44.3	33.8	0.958	− 22.5	36.2	23.5	0.029
*F*_ingress_ (intensity)^a^	16.4	49.9	46.6	< 0.001	5.0	78.8	76.3	0.077	17.5	71.0	67.5	0.004
*T*_½max_ (s)^b^	− 19.8	9.2	1.4	0.082	− 3.3	44.4	34.0	0.835	36.4	44.3	33.1	0.010
Time ratio^b^	− 57.4	1.0	0	0.849	− 377.2	48.8	39.2	0.250	− 402.8	15.4	0	0.418
*T*_max_ (s)^b^	− 9.9	9.1	1.3	0.085	4.0	44.9	34.5	0.684	− 11.7	27.1	12.5	0.094
*F*_max_ (intensity)^a^	15.4	47.9	44.5	< 0.001	4.7	78.8	76.3	0.078	15.5	68.7	64.9	0.010

*aR*^*2*^ adjusted R^2^

^a^Intensity-dependent and combined parameters with the full occlusion-step included in all analyses

^b^Time-related parameters without the full occlusion-step included in the analyses

## Discussion

Continuous quantitative ICG-FI was successfully applied using the novel, high-frequency, low-dose ICG bolus regimen. ICG-FI parameters, obtained using the PerfusionWorks^®^ software, was successfully correlated with transit time flow measurements, showing its usability for continuous quantitative assessment of tissue perfusion. The kidney was selected as study material, since the renal artery is a functional end artery, ensuring a comparable perfusion and arterial flow rate. In this study, the combined time-intensity parameter slope and the intensity-dependent parameters *F*_ingress_ and *F*_max_ were the ICG-FI parameters exhibiting the highest correlation with transit time flow probe measurements, indicating their promising applicability to detect changes in tissue perfusion under continuous perfusion assessment. In this particular study, time-related parameters did not exhibit any correlation with transit time flow measurements.

A total of 20 repeated 0.008 mg/kg ICG administrations resulted in 20 consecutive minutes of quantitative tissue perfusion assessment in each animal. The dose of 0.008 mg/kg ICG was deemed technically feasible, resulting in adequate time-intensity curves for the PerfusionWorks^®^ software analysis. When using ICG-FI to continuously monitor tissue perfusion during surgery, the ICG dose should be low enough to allow continuous bolus administration over several hours without exceeding the recommended maximum daily dosage, while simultaneously providing an adequate signal-to-noise ratio. The 0.008 mg/kg ICG dose injected for each measurement cycle would allow for 250 measurement cycles per day before reaching the current maximum recommended daily dosage of 2 mg/kg [38]. No adverse effects were observed among the animals despite the repeated injections. This is consistent with reports in another animal study using the 0.008 mg/kg ICG dosing regimen during minimally invasive surgery [20], and a clinical study using eighteen repeated injections of 0.025 mg/kg ICG to monitor autologous flap perfusion postoperatively [29]. Thus, the repeated, high-frequency, low-dose ICG bolus regimen may enable perfusion monitoring over several hours, establishing a promising and technically feasible method to continuously monitor tissue perfusion.

As evidenced by our results, traditional curve analysis falls short when no distinct time-intensity curve is present, i.e., during complete vascular occlusion (Fig. 4c). Thus, to ensure valid ICG-FI parameters, the sufficiency of the time-intensity curve should be assessed before quantification. Such an assessment was not included in the scientific version of PerfusionWorks^®^ used in the current study. In the future, the surgeon could perform this assessment before relying on the numerical output or preferably by the software prior to analysis.

The correlations between ICG-FI parameters and transit time flow measurements were higher within the respective hypo- and reperfusion phases as compared to the overall analysis, including all occlusion steps. This was interpreted as good capability of ICG-FI parameters to detect perfusion changes. In the hypoperfusion phase, the highest correlations were exhibited by *F*_ingress_ (*R*^2^ = 79%), *F*_max_ (*R*^2^ = 79%), and slope (*R*^2^ = 78%). In the reperfusion phase, the highest correlation was observed for slope (*R*^2^ = 79%), *F*_ingress_ (*R*^2^ = 71%), and *F*_max_ (*R*^2^ = 69%). Overall, slope exhibited the highest correlation (*R*^2^ = 54%). It is well-known that intensity-dependent parameters are susceptible to bias caused by inflammation, intra- and interpatient variability, light intensity, camera angulation, and working distance, while time-related parameters are less susceptible to bias [22].

In this study, time-related parameters, including *T*_ingress_, *T*_½max_, time ratio, and *T*_max_, exhibited poor correlations with flow. The poor correlation between time-related parameters and flow measurements warrants careful interpretation within the context of our experimental model. The proximity of the kidney to the aorta results in a relatively short vascular pathway compared to other organs such as the colon or extremities. This anatomical characteristic means that even during partial occlusion, the transit time for blood (and consequently ICG) to reach the renal parenchyma remains relatively stable, despite significant changes in flow volume. Additionally, the strong autoregulatory mechanisms of the kidney can maintain relatively consistent microvascular perfusion patterns despite moderate reductions in renal arterial flow. Furthermore, the renal perfusion is characterized by higher afferent blood flow than its metabolic needs [39]. These physiological characteristics likely contributed to the stability of time-related parameters in our model, even when flow was significantly reduced. This finding does not necessarily invalidate the utility of time-related parameters in other clinical contexts, particularly in organs with longer vascular pathways or different autoregulatory mechanisms. Indeed, previous studies have demonstrated the value of time-related parameters in gastrointestinal, plastic, and vascular surgery, concluding that time-related parameters appear superior to intensity-dependent parameters [22]. Instead, our results highlight the importance of considering organ-specific vascular anatomy and physiology when interpreting ICG-FI parameters. Furthermore, we find it worth mentioning, that it has been questioned whether post-revascularization ICG-FI is representative of the perfusion status of the patient [22].

On the other hand, the intensity-dependent parameters *F*_max_ and *F*_ingress_, along with the combined parameter slope, demonstrated strong correlation with flow in this study. However, integrating these parameters into the analysis necessitates either stringent standardization of experimental conditions (including imaging system, camera angulation, distance, light intensity, and dosage), or the use of relative perfusion indices to mitigate their inherent susceptibility to technical bias. Although conditional standardization seems unappealing, our study showed that it is possible to accomplish since the combined time-intensity parameter and intensity parameters exhibited strong correlation with flow. A more appealing approach, however, is the employment of a reference ROI, providing the target ROI output as a perfusion index relative to the reference ROI. The relative perfusion index has been shown to be more valid compared with the absolute interpretation, as it removes some influence of patient-specific and conditional factors [7, 26]. Furthermore, it is more feasible to interpret a perfusion index than a specific arbitrary value in a clinical setting. Relative perfusion was not applied in our study since it was unfeasible to focus the camera on both kidneys simultaneously.

Although our study investigated the continuous perfusion assessment method in an intraoperative setting the approach could be transferable to postoperative monitoring. The intraoperative approach has been criticized for showing low positive predictive value [40, 41], but conducting additional postoperative assessments may address this limitation. In free flap surgery, it has been shown feasible to measure increases in mean values per day of slope and *F*_max_ during the postoperative recovery phase [29]. Furthermore, postoperative measurements have been shown to diminish the risk of overestimating the necrosis rate, increasing the specificity of the method [42].

### Limitations

The strength of this study lies in its standardized, simple design and the utilization of transit time flow measurements as a reference, allowing for a valid and reproducible setup. The study was accomplished using a pig model with human compatible body size, anatomic, hemodynamic, and blood compositional features, establishing an important steppingstone to clinical application. However, the study was conducted in healthy animals, limited to measurements in a single organ and based on a limited sample size.

While the kidney model provided an ideal setting for controlled flow modulation and validation of our continuous ICG-FI methodology, it represents a specific physiological context that may not fully translate to all clinical applications. The kidney's short vascular pathway from the aorta and its unique autoregulatory capacity likely influenced our finding that time-related parameters did not correlate with flow changes. Time-related parameters may demonstrate different behaviors and potentially greater clinical utility in organs with longer or more complex vascular networks, such as the colon or extremities. Future studies should evaluate this methodology across organ systems with varying vascular architectures to establish parameter-specific and organ-specific reference values.

Renal vasospasms arising from surgical dissection and manipulation may have reduced renal flow in an unpredictable manner. Additionally, the results may have been influenced by movement of the flow probe during the measurements and challenges with applying a stationary occlusion with the handheld vascular clamp combined with the lack of operator blinding to transit time flow data. Although constant position of the flow probe and vascular clamp was sought maintained by mechanical fixation of clamp and probe throughout occlusion steps, it was not achievable in all experiments. Therefore, outliers were identified and excluded by visual inspection of time-intensity curves before statistical analysis, to account for significant motions artifacts. However, this approach was not feasible with regards to transit time flow data. The lack of blinding to transit time flow data was accounted for by the blinding of surgeons to ICG-FI parameter data, which were not accessed before all experiments were completed. These challenges may have introduced moderate biases.

### Perspectives

The quantitative performance of the ICG-FI needs to be accurate and reproducible to establish a useful method for assessing tissue perfusion. This study represents a significant step toward establishing a standardized and optimized clinically applicable method for continuous perfusion assessment, disclosing a novel method for providing numerical, and thus, reproducible values of perfusion, bypassing the risk of interindividual bias when surgeons are limited to visually interpret fluorescence intensity. Yet, we did not evaluate the potential detection of ischemia directly. Future studies aiming to establish reliable and procedure-specific clinical cut-off values are warranted.

While our study focused on porcine kidneys, vascularly supplied by high-flow end arteries, the technology holds potential in multiple other anatomical areas. In a recently published study, the technology was successfully applied in both the stomach, ascending colon, rectum, and spleen [20]. Another study has showed the potential of repeated ICG micro-doses regimens in reconstructive surgery, where 0.025 mg/kg ICG doses was used to predict free flap vascular complications intra- and postoperatively [29]. These regions often involve lower or more heterogeneous perfusion. We suggest the quantitative approach based on micro-dosing  to be adapted to broader surgical applications beyond the kidney, although the optimal dose may vary between subjects and anatomical areas. We expect the principle of the method, namely the continuous quantification based on repeated micro-doses of ICG, to be transferable to human surgery. We hereby present the following suggestions regarding the challenges the method faces in this context. Optimal dosing regimen for humans remains in question. Besides, further software development is needed to most precisely reflect tissue perfusion. In a clinical setting, interpreting the perfusion index is more practical than targeting a numerical value. Furthermore, the link between hypoperfusion and ischemia remains unexplored, and may be different on individual level based on comorbidities, smoking status, atherosclerosis, hemoglobin level, etc.

## Conclusion

Continuous, quantitative ICG-FI, using a novel, repeated, high-frequency, low-dose ICG bolus regimen was technically feasible and successfully detected perfusion changes (hypoperfusion, reperfusion) in the porcine kidney. In this experimental model, the combined time-intensity parameter slope and the intensity-dependent parameters *F*_ingress_ and *F*_max_ correlated well with transit time flow measurements of the right renal artery and appear promising for the continuous assessment of tissue perfusion. The lack of correlation for time-related parameters in our kidney model likely reflects organ-specific vascular characteristics rather than an inherent limitation of these parameters across all clinical applications. While slope performed best in this kidney model, both intensity-dependent and time-related parameters offer complementary insights depending on the specific organ's vascular architecture. These findings support the potential of this technology for real-time perfusion monitoring while highlighting the importance of organ-specific parameter reference values and interpretation.

## Data Availability

The datasets generated and analyzed during the current study are available from the corresponding author upon reasonable request.
